# Dr. Lee’s Objections to the Practice of Extracting the Placenta in Cases of Unavoidable Uterine Hæmorrhage

**Published:** 1846-04

**Authors:** 


15. *Dr. Lee's objections to the practice of extracting the Placenta in cases of unavoidable Uterine Hemorrhage.*—In the first case of placental presentation which came under my observation, a great gush of blood suddenly took place from the uterus, and the woman was dead before she could be seen by any practitioner. In at least twenty other cases which I have since witnessed, the first attack of hemorrhage was so sudden and profuse as to endanger life, and in several reduced the patients to a condition which rendered recovery impossible, though the most prompt and energetic treatment was employed. In all these cases the blood could not have escaped from the mother through the medium of the placenta, but from the mouths of the great veins left open in the lining membrane of the uterus by the detachment of the placenta, in consequence of which a direct communication was established between the cavity of the uterus and the cavities of the heart. If the whole mass of maternal blood contained in the cellular or cavernous structure of the placenta had flowed out in a moment, it would not have amounted to one-hundredth part of the blood which escaped from the uterus in the space of a few minutes in these cases. The cellular or cancerous structure of the placenta, which resembles a sponge, renders it impossible that any gush of blood could have taken place from the partially exposed decidual veins on the surface of the organ even if these were not constructed, as they are, like valves, and close immediately on the separation of the placental decidua from the uterus. The peculiar structure of the placenta favors the coagulation of the blood in its cells, when any portion of it is detached in the same way as hemorrhage is arrested in cut vessels by blood effused into the cellular membrane around the vessel. The cavernous structure of the portion of detached placenta is consequently invariably found to be filled with coagulated blood, and the orifices of the decidual veins covered with coagula. But the manner in which the maternal blood enters the placenta demonstrates that the great torrents of maternal blood which suddenly rush from the uterus in cases of unavoidable hemorrhage, do not proceed from the placenta, but from the vessels opened in the lining membrane of the uterus, as all the most distinguished practitioners in midwifery believe. The small curling arteries in the placental decidua, which convey the whole of the maternal blood that enters the placenta, could not possibly replenish the organ for a very considerable period if the maternal blood were entirely to escape in a few seconds from the exposed decidual veins. The fœtus would also invariably perish in cases of placental presentation, after the first attack of hemorrhage, if this were the fact, which is known to be quite the reverse.—*Medical Gazette.*

